# Continued efforts to translate diabetes cardiovascular outcome trials into clinical practice

**DOI:** 10.1186/s12933-016-0431-4

**Published:** 2016-08-11

**Authors:** Angelo Avogaro, Gian Paolo Fadini, Giorgio Sesti, Enzo Bonora, Stefano Del Prato

**Affiliations:** 1 Department of Medicine, University of Padova, Via Giustiniani 2, 35128 Padova, Italy; 2 Department of Medical and Surgical Sciences, University Magna Graecia of Catanzaro, Catanzaro, Italy; 3 Department of Medicine, Division of Endocrinology, Diabetes and Metabolism, UOC Endocrinologia, University Hospital of Verona, Verona, Italy; 4 Section of Metabolic Diseases and Diabetes, Department of Clinical and Experimental Medicine, University of Pisa, Via Paradisa, 2, 56124 Pisa, Italy

**Keywords:** Cardiovascular outcome trials, Diabetes, Complications, Treatment, Cardiovascular disease

## Abstract

Diabetic patients suffer from a high rate of cardiovascular events and such risk increases with HbA1c. However, lowering HbA1c does not appear to yield the same benefit on macrovascular endpoints, as observed for microvascular endpoints. As the number of glucose-lowering medications increases, clinicians have to consider several open questions in the management of type 2 diabetes, one of which is the cardiovascular risk profile of each regimen. Recent placebo-controlled cardiovascular outcome trials (CVOTs) have responded to some of these questions, but careful interpretation is needed. After general disappointment around CVOTs assessing safety of DPP-4 inhibitors (SAVOR, TECOS, EXAMINE) and the GLP-1 receptor agonist lixisenatide (ELIXA), the EMPA-REG Outcome trial and the LEADER trial have shown superiority of the SGLT2-I empagliflozin and the GLP-1RA liraglutide, respectively, on the 3-point MACE outcome (cardiovascular death, non-fatal myocardial infarction or stroke) and cardiovascular, as well as all-cause mortality. While available mechanistic studies largely support a cardioprotective effect of GLP-1, the ability of SGLT2 inhibitor(s) to prevent cardiovascular death was unexpected and deserves future investigation. We herein review the results of completed CVOTs of glucose-lowering medications and suggest a possible treatment algorithm based on cardiac and renal co-morbidities to translate CVOT findings into clinical practice.

## Background

Type 2 diabetes is characterized by a heavy atherosclerotic burden, inadequate compensatory remodeling and accelerated plaque progression, despite extensive use of medical therapies [1]. In diabetic patients, macrovascular and microvascular disease are tightly linked: patients with proliferative retinopathy have a 25-fold higher risk for lower limb amputation and a 2–3 fold higher risk for coronary heart disease (CHD) as compared to those without [2]. These features account for an increased cardiovascular disease (CVD) morbidity and mortality. CVD may be present at HbA1c values below the diagnostic threshold for diabetes [3], and in patients with overt type 2 diabetes, for every percentage point increase in HbA1c, the relative risk of CVD increases by about 18 % [4]. For these reasons, the latest ESC/EASD Guidelines on diabetes, pre-diabetes, and CVD emphasize the need for a stringent approach in patients with diabetes, underlying the importance of a patient-centered care [5]. In summary, in patients with type 2 diabetes, there is an excess risk and burden of CVD, which parallels the worsening of glycemic control.

Several mechanisms are though to be responsible for cardiovascular damage in diabetic patients, including hyperglycemia and oxidative stress [6], hypoglycemia [7], hyperinsulinemia and insulin resistance [8, 9]. These mechanisms can be countered by the use of different glucose-lowering medications, which are therefore expected to reduce cardiovascular risk in patients with diabetes, in addition to lowering HbA1c <7.0 %, which is still considered the goal to maximise cardiovascular benefit [10, 11]. Nonetheless, a survey from the ESC suggests that there is wide space to improve the management of patients with diabetes and cardiovascular disease [12].

We herein aim to briefly re-analyse the results of randomized controlled trials (RCTs) reporting the effects of various glucose-lowering medications on cardiovascular outcomes in patients with type 2 diabetes. To this end, we run a PubMed search for RCTs with the following terms: “type 2 diabetes” “randomized controlled trial” “cardiovascular” and screened for cardiovascular outcome trials (CVOTs) assessing safety or efficacy of glucose-lowering medications.

## The macrovascular paradox

While there is much evidence that the risk of CVD increases along with HbA1c, intervention trials aimed at determining whether tight glycemic control is associated with a reduction in CVD have offered controversial results (meta-analyzed in [13]). This is reflected, for example, by the observation that there is no correlation between the glycaemic control and coronary vascular function in diabetic patients [14]. We call this the “macrovascular paradox”: i.e. the failure to reduce macrovascular complications to the same extent as the microvascular complications, despite comparable reductions in HbA1c. A number of algorithms have been proposed to ensure glycemic control whenever lifestyle measurements fail to keep HbA1c at target. The choice of drugs in the treatment strategy is mainly based on efficacy, risk of hypoglycaemia, effect on body weight, other side effects and, ultimately, costs. Effect on CVD risk is an important item that may guide drug selection. Multiple reasons have been claimed to potentially account for the negative findings of CVD reduction trials, including: drug-induced hypoglycemia, weight gain, other side effects, wrong HbA1c target, short duration of the trials. A likely explanation for the CVD paradox may rely on the multifactorial nature of the CVD risk in diabetes, as highlighted by the results of the STENO-2 study [15]. As a corollary of this, pharmacologic agents with effects exceeding the glucose lowering action may be expected to confer either negative or positive impacts on CVD.

## Trials on cardiovascular effects of glucose lowering agents

Several articles have already extensively reviewed pre-clinical and clinical findings on the cardiovascular effects of glucose-lowering medications [16–21]. We herein focus on data coming from early trials and from so-called cardiovascular outcome trials (CVOT) requested by regulatory agencies for marketing authorization approval [22].

Metformin is widely accepted as the first-choice agent for glucose lowering largely because of the results of the UKPDS sub-study showing a significant 39 % reduction in myocardial infarction (MI) in a limited number of overweight diabetic patients. After 10 years of the UKPDS post-trial monitoring, a significant reduction in nonfatal MI was observed in patients in the intensive arm (initially treated with insulin or sulfonylureas) [23]. However, metformin use has been recently questioned on the basis of both efficacy and outcomes [24]. In the UKPDS, the SU use, despite significant increase in hypoglycemia, was not associated to an increase in MI fatality compared to no SU use [25]. The action in diabetes and vascular disease: preterax and diamicron modified release controlled evaluation (ADVANCE) trial showed that intensive glycemic control based on gliclazide modified release reduced the incidence of combined major macro- and microvascular events, primarily because of a reduction in the incidence of new or worsening nephropathy [26]. In the Outcome Reduction With an initial glargine intervention (ORIGIN) trial, early use of basal insulin to achieve normal fasting plasma glucose levels had no effect on CVD outcomes compared with guideline-suggested glycemic control [27].

In the STOP-NIDDM trial, acarbose was initially suggested to reduce cardiovascular risk in patients with impaired glucose tolerance [28]. Furthermore, a meta-analysis of long term studies concluded that a similar effect may be present in type 2 diabetes, but the number of patients was very small and the conclusion of the analysis was very controversial and not reproducible [29–31].

An additional option in treatment algorithms is the use of thiazolidinediones. In the Prospective Pioglitazone Clinical Trial in Macrovascular Events (PROactive), pioglitazone, a PPAR-gamma agonist, when added to baseline anti-hyperglycemic regimen, had no apparent benefit on a broad, combined, primary end point [32]. However, a pre-specified secondary outcome (MI, stroke, and cardiovascular mortality) was reduced by 16 %, in spite of an increase in heart failure (HF). A different tale is known for rosiglitazone, which has been implicated in an increase risk of MI [33, 34], although most recent analyses have casted doubts on the initial data interpretation [35]. Negative CVD outcomes have been reported with the use of dual PPAR alpha-gamma agonists Tesaglitazar and Muraglitazar [36].

## The lesson of the cardiovascular outcome trials

In 2008, following the withdrawal of rosiglitazone from the market because of potential negative impact on CVD outcomes [33, 34], the Food and Drug Administration (FDA) issued guidance on the assessment of CVD risk for all new drugs to treat type 2 diabetes [22]. Following this, a large number of patients with type 2 diabetes have been enrolled in CV outcome trials (CVOT, summarized in Table 1). We now have the results of 6 of such trials: 3 assessing safety of dipeptidyl peptidase (DPP) 4 inhibitors [37–39], 2 testing the safety of a glucagon-like peptide-1 receptor agonist (GLP1-RA) [40, 41], and 1 of an inhibitor of the sodium-glucose co-transporter (SGLT)-2 [42]. All DPP4-I CVOTs met the safety primary endpoint of non-inferiority versus placebo with respect to 3-point MACE (CVD mortality, non-fatal myocardial infarction, and non-fatal stroke). In the Saxagliptin Assessment of Vascular Outcomes Recorded in Patients with Diabetes Mellitus (SAVOR) trial, a statistically significant increase in hospitalization for HF was observed in the patients randomized to saxagliptin, although no increase in CVD mortality occurred in these individuals. This was not observed in CVOTs assessing sitagliptin and alogliptin [38, 39]. The issue of hospitalization for heart failure has been subsequently analyzed in several observational trials [43] and meta-analyes [44], most concluding for a neutral effect of DPP4-I on heart failure risk.

**Table 1:** Characteristics of CVOTs

	SAVOR	TECOS	EXAMINE	ORIGIN	ELIXA	LEADER	EMPA-REG
Trial characteristic							
Drug	Saxagliptin	Sitagliptin	Alogliptin	Glargine	Lixisenatide	Liraglutide	Empagliflozin
Comparator	Placebo	Placebo	Placebo	Placebo	Placebo	Placebo	Placebo
No. patients	16492	14671	5380	12537	6068	9340	7020
Duration, years	2.1	3.0	1.5	6.2	2.1	3.8	3.1
Primary endpoint	3-point MACE	4-point MACE	3-point MACE	3-point MACE	3-point MACE	3-point MACE	3-point MACE
Major secondary endpoint	3-point MACE + hospitalization for unstable angina, coronary revasc. or HHF	3-point MACE	3-point MACE + urgent revasc. for unstable angina	3-point MACE + revasc. or HHF (co-primary)	3-point MACE + HHF or revasc.	3-point MACE + coronary revasc. or hospitalization for unstable angina or HHF	4-point MACE
Pts characteristics							
Age, years (mean ± SD)	65.0 ± 8.5	65.5 ± 8.0	61.0 (median)	63.6 ± 7.8	60.3 ± 9.6	64.3 ± 7.2	63.1 ± 8.7
Diabetes duration, years	10.3 (IQR 5.2–16.7)	11.6 ± 8.1	7.2 (IQR 2.7–13.7)	5.4 ± 6.0	9.3 ± 8.2	12.8 ± 8.1	57.4 % > 10 years
Baseline HbA1c	8.0 ± 1.4	7.2 ± 0.5	8.0 ± 1.1	6.4 (IQR 5.8–7.2)	7.6 ± 1.3	8.7 ± 1.5	8.1 ± 0.8
Baseline BMI	31.1 ± 5.6	30.2 ± 5.6	28.7 (IQR 5.6–68.3)	29.8 ± 5.2	30.2 ± 5.7	32.5 ± 6.3	30.7 ± 5.3
Insulin users, %	41.4	23.2	29.9	0	39.1	44.5	48.3
% with CVD	78.5	74.0	100	58.9	100	81.3	75.6 (CAD)
% with eGFR <60 ml/min/1.73 m^2^	15.6	9.4 % (<50 ml/min/1.73 m^2^)	29.1	N/A	23.2	23.1	26.0
Annual event rate in placebo arm, %	3.5	3.8	7.9	2.9	6.3	3.9	4.4
CV outcome							
HR primary endpoint (95 % C.I.)	1.00 (0.89–1.12)	0.98 (0.88–1.09)	0.96 (≤1.16)	1.02 (0.94–1.11)	1.02 (0.89–1.17)	0.87 (0.78–0.97)*	0.86 (0.74–0.99)*
HR secondary endpoint (95 % C.I.)	1.02 (0.94–1.11)	0.99 (0.89–1.11)	0.95 (≤1.14)	1.04 (0.97–1.11)	0.97 (0.85–1.10)	0.88 (0.81–0.96)	0.89 (0.78–1.01)
HR HHF (95 % C.I.)	1.27 (1.07–1.51)*	1.00 (0.83–1.20)	1.07 (0.79–1.46)	0.90 (0.77–1.05)	0.96 (0.75–1.23)	0.87 (0.73–1.05)	0.65 (0.50–0.85)*
HR CV death (95 % C.I.)	1.03 (0.87–1.22)	1.08^a^	0.79 (0.60–1.04)	1.00 (0.89–1.13)	0.93^a^	0.68 (0.66–0.93)	0.62 (0.49–0.77)*
HR any death (95 % C.I.)	1.11 (0.96–1.27)	1.03^a^	0.88 (0.71–1.09)	0.98 (0.90–1.08)	0.94 (0.78–1.13)	0.85 (0.74–0.97)	0.68 (0.57–0.82)*
NNT primary endpoint (3 years)	N/A	N/A	N/A	N/A	N/A	66	61
NNT death (3 years)	N/A	N/A	N/A	N/A	N/A	98	39
Efficacy							
HbA1c change, %	−0.3*	−0.3*	−0.36*	−0.3*	−0.4*	−0.4*	−0.3*
Body weight change, kg	−0.4	N/A	Neutral	+1.1*	−0.6*	−2.3*	−1.4*
Renal endpoints	Albuminuria improved	No effect	No effect	No effect	Lower increase in albuminuria	Lower rate of nephropathy events	Lower progression of CKD

Though the ORIGIN trial is not strictly a CVOTs it has been included as being one of the milestone mega-trial in this field

*MACE* major adverse cardiovascular events, *HHF* hospitalization for heart failure, *Revasc*. revascularization, *IQR* interquartile range, *NNT* number needed to treat. N/A, not available

* p < 0.05

^a^Extrapolated from crude data

In the evaluation of lixisenatide in acute coronary syndromes (ELIXA) trial, the use of lixisenatide in diabetic patients with a recent acute coronary syndrome showed neutrality on CVD outcomes with no increase in the risk of heart failure hospitalization [40].

Both DPP4-I and GLP-1RA have been integrated in treatment algorithms before the results of these trials were published/disclosed, mainly because of the favorable efficacy-safety profile. With the question remaining open with respect to the increased risk of hospitalization for heart failure reported in SAVOR trial (not confirmed in any other study), overall evidence is available for safe use of DPP4-I across populations with different degree of CVD risk, including those with recent acute coronary syndrome [38]. Though these results have been welcome as reassuring, the diabetes community continues asking whether these treatments may, under different circumstances, lend to some degree of CVD protection.

## EMPA-REG Outcome trial and LEADER trial

Results of the two latest CVOTs, namely the EMPA-REG Outcome trial [42] and the Liraglutide effect and action in diabetes: Evaluation of cardiovascular outcome results (LEADER) trial [41] have recently stirred much enthusiasm. In these trials, as in SAVOR, TECOS and ELIXA, diabetic patients at very high CVD risk, were enrolled to determine the CVD safety of the SGLT2 inhibitor empagliflozin and the GLP-1RA liraglutide.

In the EMPA-REG Outcome trial, the primary outcome was a composite of death from CV causes, nonfatal myocardial infarction, and nonfatal stroke (3-point MACE). Two daily doses of the drug were tested: 10 and 25 mg. As compared to placebo, empagliflozin (pooled analyses of 10 and 25 mg) showed non-inferiority for 3-point MACE, non-inferiority for 4-point MACE (including hospitalization for unstable angina), superiority for 3-point MACE and not for 4-point MACE. With respect to secondary endpoints, patients randomized to empagliflozin had significantly reduced risk of hospitalization for HF by 35 %, reduced risk CV death by 38 %, and reduced risk for all-cause mortality by 32 %. The mechanisms responsible for these results are still unclear, being possibly related to pleiotropic effects on risk factors, to hemodynamic effects, and possible direct effects on the heart and vasculature [45, 46]. Furthermore, empagliflozin, compared to placebo, significantly slowed progression of kidney disease and loss of glomerular filtration over time in high-risk patients of the EMPA-REG Outcome trial [47].

In the LEADER trial, the primary endpoint was the same as in the EMPA-REG Outcome trial [41]. In the primary data set and in per protocol analyses, compared to placebo, liraglutide significantly reduced occurrence of the 3-point MACE by 13 %, cardiovascular death by 22 %, and all-cause mortality by 15 %, without significant effects on non-fatal MI, non-fatal stroke and hospitalization for heart failure [41]. These findings appear to some extent similar to what observed in the EMPA-REG Outcome trial, and occurred in parallel with mild reductions in body weight and systolic blood pressure [41].

In EMPA-REG, the early and unusual divarication of mortality curves deserves attention. Unlike in the LEADER, such a rapid effect suggests treatment has little effect on the atherosclerotic process pointing for an effect unlikely to be mediated through the modulation of glucose or lipid metabolism. Similarly, the modest reduction of body weight, is unlikely to account for the reported effect. More interesting is the effect on blood pressure, although prior trials using blood pressure lowering drugs have shown a positive effect on CVD outcome to occur at a later time than in EMPA-REG [48]. A minor impact on the atherosclerotic process is also supported by the lack of any significant effect on nonfatal MI and stroke. These results suggest that the use of empagliflozin does not necessary protect from the CV event, rather with the mortality linked to the event itself.

Of note, the early separation of the mortality curves is paralleled by an even earlier divarication of the curves for hospitalization for heart failure, suggesting at least part of the beneficial effect of empagliflozin to be exerted through volume depletion: in keeping with this, a 4 % hematocrit increase was recorded in the empagliflozin treated patients [49]. Ferrannini et al. have speculated on the mechanisms at work suggesting that a switch to fatty acid utilization, concurrent with better oxygen delivery to the tissues, cooperates with small changes in body weight and blood pressure to achieve cardioprotection by SGLT2 inhibition [50].

In the LEADER trial, survival curves for 3-point MACE and mortality separate later (since about 12–18 months from randomization), and there were no effect on heart failure. This observation suggests that liraglutide, differently from empagliflozin, may reduce the occurrence of cardiovascular events mainly by preventing progression of atherosclerosis, possibly thanks to a better control of risk factors and despite a mild rise in heart rate [41]. This view is supported by a wealth of pre-clinical and pathophysiologic studies (reviewed in [17]).

While we wait for additional studies able to clarify which mechanism(s) can explain the improvement in CVD outcomes by empagliflozin and liraglutide, the clinical implications of these results need to be critically put in the perspective of current guidelines, treatment algorithms, and health care economy. With respect to this, a number of questions need to be addressed: 1. Can the results obtained with empagliflozin and liraglutide extended to other drugs of the same class? By now, of the two GLP-1RA evaluated in CVOTs, only liraglutide, but not lixisenatide, achieved cardioprotection. Before concluding for a drug-specific effect, differences in the patient populations and study design between ELIXA and LEADER trials should be taken into account (Table 1), whereas results of ongoing trials with other GLP-1RAs will help concluding on a class-effect. 2. As the proportion of patients with established CVD was high in the EMPA–REG (75.6 % had CAD) and LEADER (81.3 % had CVD), can the results obtained be translated to other patient categories, i.e. patients without established CVD? 3. Is there a specific sub-population that may derive a specific benefit from the treatment with SGLT2-I and GLP-RA? Answering these questions will require further analysis of the EMPA-REG and LEADER databases as well as more specific clinical and mechanistic studies. So far, in both the EMPA-REG Outcome trial and the LEADER trial, there is a signal indicating that patients with renal impairment are those who benefited most from treatment with empagliflozin and liraglutide, respectively, as both drugs appear to reduce kidney-related endpoints [41, 47]. Yet, initiating therapy with empagliflozin is still not recommended in patients with eGFR <60 ml/min/1.73 m^2^. While this limitation may change in the near future, liraglutide can already be used in patients with stage III CKD, being not indicated in patients with eGFR <30 ml/min/1.73 m^2^.

Results of the CVOT with canagliflozin (CANVAS) and dapagliflozin (DECLARE) will shed further light on the results obtained with empagliflozin, possibly showing whether the beneficial effects shown in the EMPA-REG outcome trial can be extended to the SGLT2-inhibitor class and to individuals with a different CV risk profile [21].

Yet, a 22 and 38 % risk reduction of CVD mortality observed with liraglutide and empagliflozin, respectively, is too strong to be overlooked, and will likely require a favorable revision of the positioning of these drugs in the current treatment algorithm of type 2 diabetes. In order to do so, some features of the EMPA-REG and LEADER trials are worth a consideration. First of all, the results of the trial pertain to a well-defined diabetic population, i.e. patients with established prior CV events. As such, it cannot be generalized to the wide spectrum of clinical diabetes; in this context, particularly striking have been the superiority of the primary outcome in patients with an age ≥65 years, and the death for CV causes in the group with body mass index <30 kg/m^2^.

## Comorbidities-driven treatment

In patients with type 2 diabetes, especially in the elderly, the presence of CVD is central, with emphasis on concomitant heart failure and chronic kidney disease (CKD). In obese patients, treatment should aim at improving glycemic control and reducing body weight. Although evidence for the CV protection of metformin is rather limited, the drug has become quite familiar after more than 50 years of use, has advantageous cost effectiveness and a modest lowering effect on body weigh to remains the preferred background treatment.

If the patient has asymptomatic CVD or prior MACE and eGFR ≥60 ml/min/1.73 m^2^ and heart failure NYHA class I–III, metformin plus empagliflozin/liraglutide should be considered as these are the typical patients included in the EMPA-REG Outcome and LEADER trials, with liraglutide currently being usable in stage III CKD. For patients with no sign of heart failure, either pioglitazone (or DPP4-I) may represent a therapeutic option. For patients with a eGFR of 60–30 ml/min/1.73 m^2^, liraglutide should be consider the preferred choice. Though 26 % of patients in the EMPA-REG Outcome trial fell in this eGFR category and they benefited most in terms of cardiovascular protection, initiation of empagliflozin is currently discouraged in stage III CKD, and lower glycemic effect is expected. DPP4-I can be used even with an eGFR <30 ml/min/1.73 m^2^ because of their overall safety and efficacy, granted dose adjustment is made for the compounds requiring it.

If the patient has evidence of CKD without heart failure, pioglitazone can be a reasonable option as shown in a subanalysis of the PROactve trial [51]. Insulin and SU, if needed, should be used with caution because of potential risk of hypoglycaemia, for the latter gliclazide modified release may be a preferred choice because of the available data obtained in ADVANCE [26]. The ORIGIN trial has clearly demonstrated cardiovascular safety of insulin glargine, and the risk of hypoglycemia with basal insulin is lower than during basal-bolus regimen. However, as glargine and liraglutide show similar glycemic effects [52, 53], the latter should be preferred for the lower hypoglycemia risk [54] and in view of the LEADER trial [41], except for eGFR <30 ml/min/1.73 m^2^. In general terms, use of insulin has been shown to be effective even in the log run in patients with acute coronary syndrome in the DIGAMI 1 trial [55, 56], though superiority of insulin over standard care was not confirmed the DIGAMI 2 [57].

In patients without CVD, a SGLT2-I or a GLP-1RA should be considered if body weight loss is required, whereas a DPP4-I can be considered whether when weight neutrality is sought. In this context, incretin-based therapy has greater HbA1c reduction if patients have obesity/metabolic syndrome with a greater effect for GLP-1RA as compared to DPP4-I [58]. Pioglitazone could be considered if obesity is associated with evidence for marked insulin resistance as supported by the co-existence of dyslipidemia, inflammatory markers, and subclinical CVD [59, 60].

**Fig. 1:**
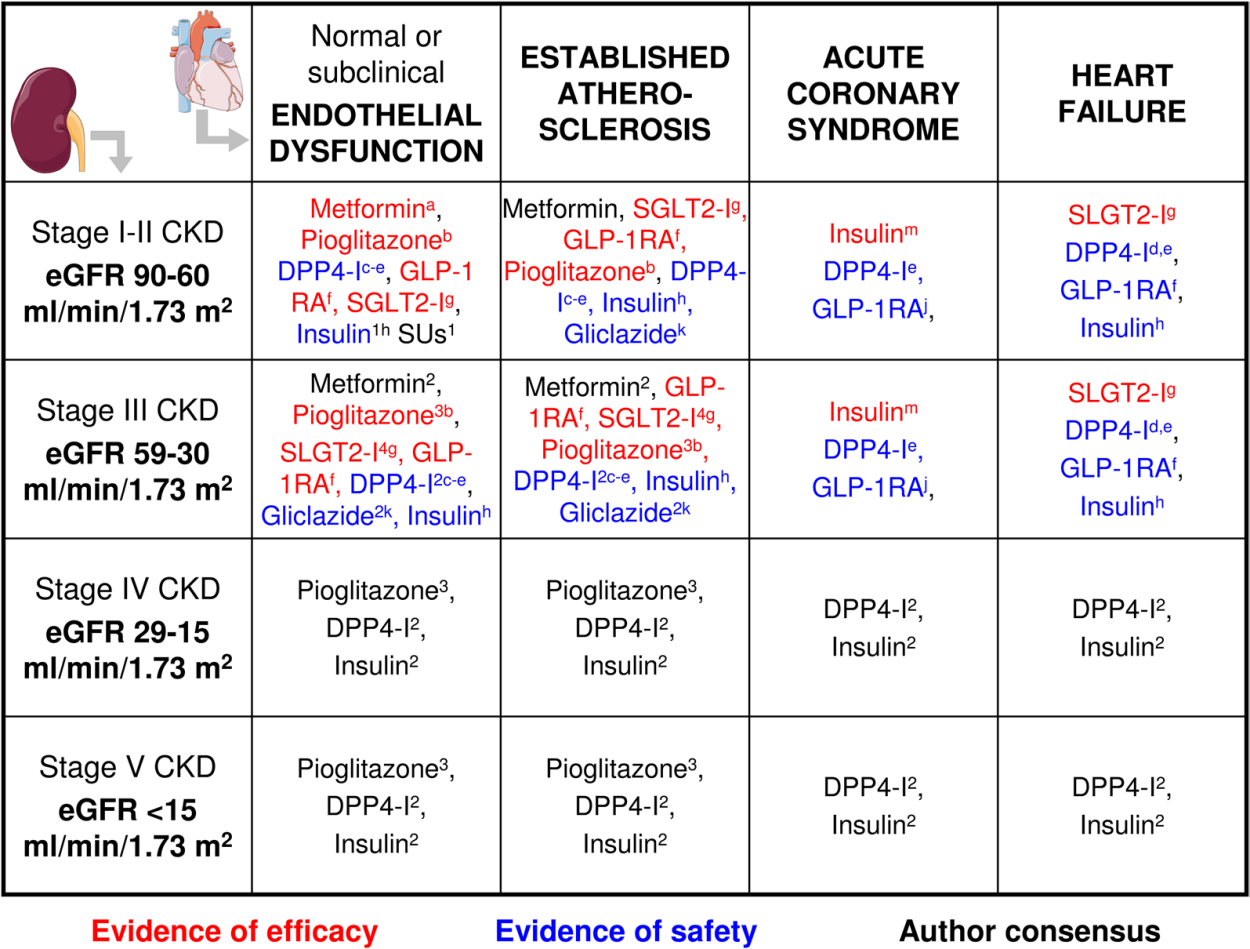
A treatment algorithm based on cardiac and renal co-morbidities and CVOTs. ^1^To be used with caution because of the risk of hypoglycemia; ^2^consider dose reduction (except for linagliptin) and monitor eGFR frequently; ^3^preferred in the presence of marked insulin resistance; ^4^initiation of therapy currently not recommended. ^a^UKPDS; ^b^PROACTIVE trial; ^c^SAVOR; ^d^TECOS, ^e^EXAMINE; ^f^LEADER trial; ^g^EMPA-REG Outcome trial; ^h^ORIGIN trial; ^k^ADVANCE; ^j^ELIXA; ^m^DIGAMI 1

In non-obese or mildly obese patients, the prevention of body weight gain may represent an important target along with glycemic control. In this case, a DPP4-I can represent a good choice due to its weight neutrality and the overall and CVD safety. In the case of leaner patients, additional treatment options may be gliclazide modified release and insulin. The former has been shown to significantly decrease new or worsening nephropathy with relatively little risk for severe hypoglycemia [26], while CV safety of basal insulin in the early stage of diabetes is supported by the results of the ORIGIN study [27]. It must be mentioned that in many patients, triple (if not quadruple) oral or oral plus injective treatment becomes necessary during the course of the disease: this implies subsequent decisions about drug combinations. Nowadays choices are multiple and it should always be carefully considered the many aspects of clinics phenotype as suggested by the ADA/EASD guidelines. This makes the proposition of a more stringent algorithm difficult (Fig. 1).

## Limitations

**Fig. 2:**
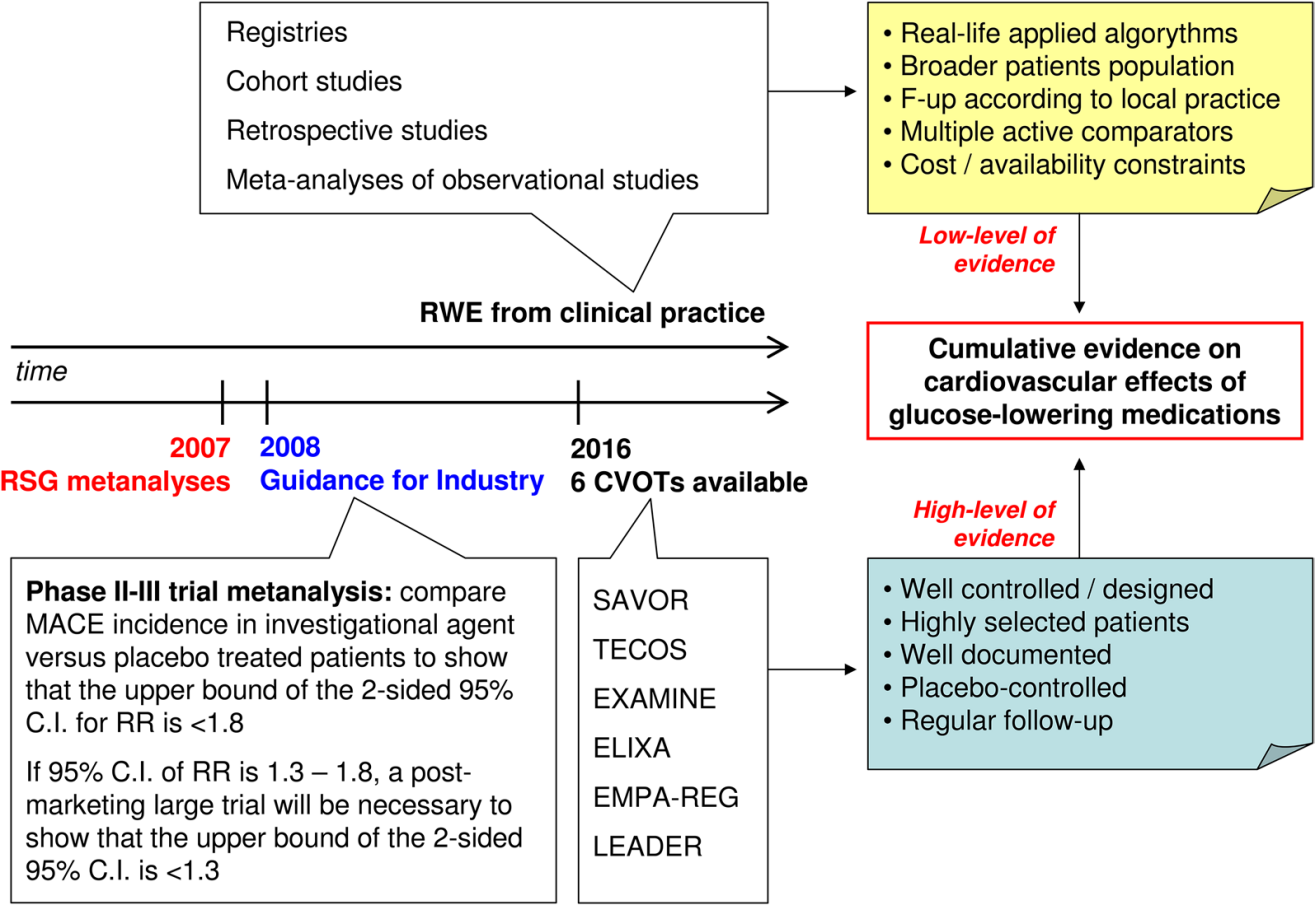
The interplay between data derived from CVOTs and real world evidence for assessing the cardiovascular effects of glucose-lowering agents. *RSG* rosiglitazone, *CVOTs* cardiovascular outcome trials, *RWE* real world evidence, *RR* risk ratio

The view presented in this article is largely based on results from RCTs. It should be noted that, although such mega-trials provide the highest level of evidence, they have intrinsic characteristics that limit their generalizability and transferability to clinical practice. We have already mentioned that the study population in most CVOTs is very different from the entire population of patients who are entitled to receive the respective medication. In fact, RCTs differ from clinical practice in several instances. For this reason, there is great interest in real world evidence (RWE) on glucose-lowering medications and how these fit with results from RCTs (Fig. 2). Data from RWE studies can complement RCTs, but they provide a lower level of evidence and can yield quite different results. This has been clearly shown for the risk of hospitalization for heart failure in DPP-4i treated patients, where meta-analyses of RCTs and observational studies [43] can reach to different conclusions [44]. Nonetheless, we endorse the importance of RWE in the evaluation of glucose-lowering medications, especially to explore aspects that cannot be extracted from RCTs. For instance, placebo-controlled RCTs may be poorly informative for clinical practice because they do not provide a comparative assessment of different glucose-lowering medications against cardiovascular outcomes, whereas RWE data can evaluate complex regimens against cardiovascular risk [61].

In addition, we would like to underline that the pharmaco-centric view presented here ignores the importance of a healthy lifestyle and a comprehensive approach to prevent cardiovascular disease, as originally shown in the STENO-2 study [15], and confirmed by the analysis of treatment-dependent and -independent factors associated with cardiovascular morbidity [62], as well as by the effects of a multidisciplinary risk assessment and management program [63]. Although the Look-AHEAD study found no cardiovascular benefit of an intensive lifestyle intervention that promoted weight loss [64], there are still reasons to recommend lifestyle changes in type 2 diabetes mellitus and obesity since early disease stages [65]. Finally, bariatric surgery, another non-pharmacologic approach, may reduce cardiovascular risk in obese people with type 2 diabetes, as shown by recent meta-analyses of observational studies [66, 67].

## Conclusions

The most recent CVOTs have expanded our knowledge on the potential effects of glucose-lowering agents on CVD risk. Though most of them have proven CVD safety, the EMPA–REG Outcome trial and LEADER trial have provided evidence for significant improvement of CVD outcomes. While we do not yet have firm explanations for the mechanisms accounting for the observed beneficial effect or whether any specific population may benefit more (for instance patients with heart failure or CKD), it sounds legitimate to try putting these observations in the perspective of current treatment algorithms. We believe this exercise is needed to avoid inappropriate over-use of SGLT2-I and GLP-1RA, before all needed information is gathered while ensuring they are used in keeping with the available evidence. Central to our proposal is the presence of CVD. Patient’s phenotype, degree of renal function, presence of heart failure, allows for a further patient’s population breakdown for more appropriate pharmacologic treatment selection. The results of the EMPA-REG and LEADER trials mostly support the use of empagliflozin or liraglutide in patients who have established CVD, a prior MACE, with or without stage I–III CKD, but we still do not know whether similar positive effects should be extended to the other drugs of the same classes.

The prevention of CVD complications and the safe treatment of patients who already have suffered a CVD event, especially in the elderly patients, remain a major task in treating type 2 diabetes. CVOTs and RWE data represent the basis for evidence-based treatment though we must acknowledge this is a moving target as results of new and ongoing trial will be released requiring a constant revision of treatment algorithms.

## Abbreviations

ADA: American Diabetes Association
ADVANCE: action in diabetes and vascular disease: preterax and diamicron modified release controlled evaluation
CAD: coronary artery disease
CHD: coronary heart disease
CKD: chronic kidney disease
CVD: cardiovascular disease
CVOT: cardiovascular outcome trial
DIGAMI: diabetes and insulin-glucose infusion in acute myocardial infarction
DPP4: dipeptydil peptidase 4
EASD: European Association for the Study of Diabetes
eGFR: estimated glomerular filtration rate
ELIXA: evaluation of lixisenatide in acute coronary syndromes
EMPA-REG: empagliflozin cardiovascular outcome event trial in type 2 diabetes mellitus patients
ESC: European Society of Cardiology
GLP-1: glucagon-like peptide 1
HF: heart failure
LEADER: liraglutide effect and action in diabetes: evaluation of cardiovascular outcome results
MACE: major adverse cardiovascular events
MI: myocardial infarction
NYHA: New York Heart Association
ORIGIN: outcome reduction with an initial glargine intervention
PPAR: peroxisome proliferator activating receptor
PROactive: prospective pioglitazone clinical trial in macrovascular events
RCT: randomized clinical trial
RWE: real world evidence
SAVOR: the saxagliptin assessment of vascular outcomes recorded in patients with diabetes mellitus
SGLT2: sodium-glucose cotransporter 2
STOP-NIDDM: stop non insulin dependent diabetes mellitus
SU: sulphonylurea
TECOS: Sitagliptin Cardiovascular Outcomes Study
UKPDS: United Kingdom Perspective Diabetes Study
